# Modeling Optimal Clinical Thresholds for Elective Abdominal Hernia Repair in Patients With Cirrhosis

**DOI:** 10.1001/jamanetworkopen.2022.31601

**Published:** 2022-09-13

**Authors:** Nadim Mahmud, David S. Goldberg, Samir Abu-Gazala, James D. Lewis, David E. Kaplan

**Affiliations:** 1 Division of Gastroenterology and Hepatology, Perelman School of Medicine, University of Pennsylvania, Philadelphia; 2 Department of Medicine, Corporal Michael J. Crescenz VA Medical Center, Philadelphia, Pennsylvania; 3 Center for Clinical Epidemiology and Biostatistics, Department of Biostatistics, Epidemiology & Informatics, Perelman School of Medicine, University of Pennsylvania, Philadelphia; 4 Leonard David Institute of Health Economics, University of Pennsylvania Perelman School of Medicine, Philadelphia; 5 Division of Digestive Health and Liver Diseases, Department of Medicine, University of Miami Miller School of Medicine, Miami, Florida; 6 Division of Transplant Surgery, Perelman School of Medicine, University of Pennsylvania, Philadelphia

## Abstract

**Importance:**

Patients with cirrhosis have increased risk of postoperative mortality. Several models have been developed to estimate this risk; however, current risk estimation scores cannot compare surgical risk with the risk of not operating.

**Objective:**

To identify clinical optimal thresholds to favor operative or nonoperative management for a common cirrhosis surgical scenario, the symptomatic abdominal hernia.

**Design, Setting, and Participants:**

This was a Markov cohort decision analytical modeling study evaluating elective surgery vs nonoperative management for a symptomatic abdominal hernia in a patient with cirrhosis. Transition probabilities and utilities were derived from the literature and from data using an established cirrhosis cohort in the Veterans Health Administration. Participants included patients who were referred to a surgery clinic for a symptomatic abdominal hernia. Data were obtained from patients diagnosed with cirrhosis between January 1, 2008 and December 31, 2018. Data were analyzed from January 1 to May 1, 2022.

**Main Outcomes and Measures:**

Expected quality-adjusted life-years (QALYs) were estimated for each pathway and iterated over baseline model for end-stage liver disease–sodium (MELD-Na) scores ranging from 6 to 25. Markov models were cycled over a 5-year time horizon.

**Results:**

A total 2740 patients with cirrhosis (median [IQR] age, 62 [56-66] years; 2699 [98.5%] men) were referred to a surgery clinic for a symptomatic abdominal hernia; 1752 patients (63.9%) did not receive surgery. The median (IQR) follow-up was 42.1 (25.3-70.0) months. Using this cohort to estimate the mortality risk of operative and nonoperative pathways, an initial MELD-Na threshold of 21.3 points, below which surgery was associated with maximized QALYs was identified. Nonoperative management was associated with increased QALYs above this MELD-Na threshold. Although more patients experienced death with a surgical treatment decision across all initial MELD-Na values, this was counterbalanced by increased time spent in a resolved hernia state associated with increased utility. Model results were sensitive to the probability of hernia recurrence and hernia incarceration and utility decrement in the symptomatic hernia state.

**Conclusions and Relevance:**

This decision analytical model study found that elective surgical treatment for a symptomatic abdominal hernia was favored even in the setting of relatively high MELD-Na scores. Patient symptoms, hernia-specific characteristics, and surgeon and center expertise may potentially impact the optimal strategy, emphasizing the importance of shared decision-making.

## Key Points

**Question:**

What are the optimal clinical thresholds for pursuing elective hernia repair in patients with cirrhosis with symptomatic abdominal hernias?

**Findings:**

This decision analytical model study in a large cohort 2740 patients with cirrhosis identified a model for end-stage liver disease–sodium score threshold of 21.3 below which elective surgical treatment was favored over nonoperative management.

**Meaning:**

These findings suggest that elective surgical hernia repair may be favored even in patients with advanced severity of liver disease, contrary to common practice.

## Teaser

This decision analytical model study uses Markov modeling to identify optimal clinical thresholds for operative vs nonoperative management of symptomatic abdominal hernia in patients with cirrhosis.

## Introduction

As the cirrhosis burden in the US increases,^1^ so has the volume of surgical treatments for cirrhosis.^2^ Preoperative risk stratification has been challenging owing to myriad contributors of cirrhosis to surgical risk, such as impaired synthetic function, malnutrition and frailty,^3,4^ portal hypertension,^5,6^ and deranged hemostasis.^7,8^ A novel cirrhosis surgical risk score, the VOCAL-Penn Score (VPS), improves estimation of short-term mortality compared with the model for end-stage liver disease–sodium (MELD-Na) score, Child-Turcotte-Pugh (CTP) score, and the Mayo Risk Score (MRS).^9^ Superior performance of the VPS was confirmed in 2 independent health systems,^10^ and a subsequent VPS model to estimate postoperative decompensation has also been published.^11^

For patient counseling and shared decision-making, patients (and clinicians) must understand the risks and benefits of potential treatment pathways. A limitation of existing risk estimation scores is that they cannot contextualize the risk of operative vs nonoperative management. This is because estimation scores, such as the MRS or VPS, were derived exclusively from cohorts of patients who underwent surgical treatment.^9,12^ As with the risks associated with surgical treatment, estimating the morbidity and mortality risk of the nonoperative alternative must incorporate both the severity of underlying liver disease and the indication for surgical treatment. The goal of this study was to focus on a commonly encountered surgical scenario in patients with cirrhosis, the symptomatic abdominal hernia, and use decision analysis methods to identify when operative vs nonoperative management would be favored.

## Methods

This decision analytical model study received institutional review board approval from the Corporal Michael J. Crescenz Philadelphia Veterans Affairs Medical Center. The requirement for informed consent was waived under the Common Rule (45 CFR 46.116). This study is reported following the Consolidated Health Economic Evaluation Reporting Standards (CHEERS) reporting guideline.

### Markov Model and State Transition Diagram

**Figure 1.**
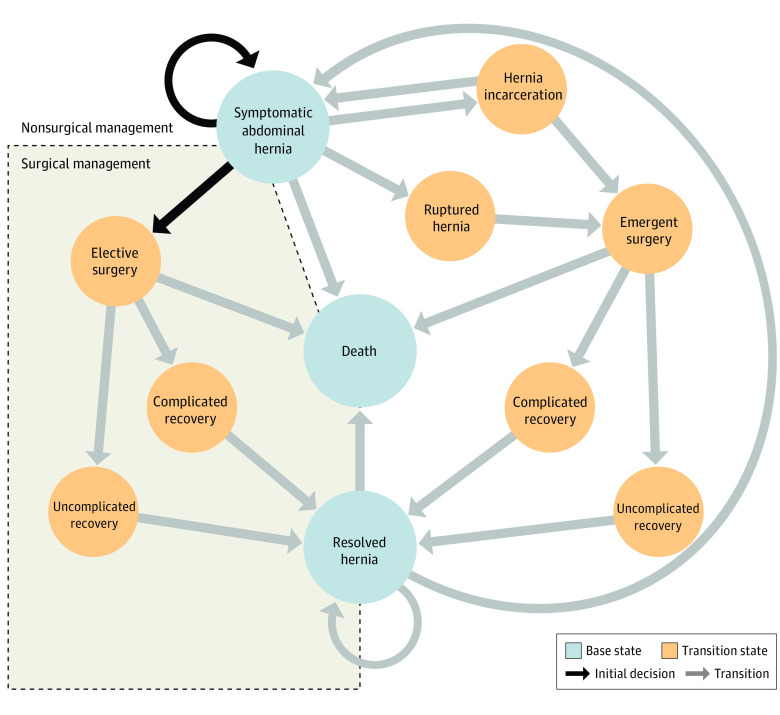
Markov State Transition Diagram

We performed a Markov cohort decision analysis for an elective surgical scenario in a patient with cirrhosis with a symptomatic abdominal hernia. This approach entails an initial clinical decision (nonsurgical vs surgical management) followed by iteration through Markov cycles (eFigure 1 in the Supplement). The Markov state-transition diagram in Figure 1 illustrates possible base states and transition states that a patient may experience during 1 Markov cycle after the initial management decision. This model assumes 3 possible base states: symptomatic abdominal hernia, resolved hernia, and death. During a given Markov cycle, a patient progresses through transition states dictated by transition probabilities before coming to rest at a base state. Base states result in positive utility increments during a Markov cycle (with the exception of death, which has a utility of 0), and all utilities associated with transition states result in a decrement of utility (eg, a patient incurs a negative utility for experiencing a ruptured hernia and emergency surgery). Each Markov cycle was 180 days to align with established risk estimation tools and plausible time horizons of adverse outcomes related to surgical treatment. Estimation of transition probabilities leading to death was accomplished through isolation of a cohort of patients with cirrhosis in the Veterans Health Administration (VHA) who were referred to surgery clinic for a symptomatic abdominal hernia (the VOCAL-VASQIP cohort).^9,11^ Among patients who did not receive surgical treatment, the VPS estimated the probability of 180-day mortality in elective and emergent operative scenarios, and a logistic regression model fit with the MELD-Na score estimated the probability of 180-day mortality for nonoperative management. Further details, including sources for additional transition probabilities and utilities, are provided in Table 1 and the eMethods in the Supplement. Patient race and ethnicity were determined from self-reported fields in the electronic medical record and categorized as Asian, Black, Hispanic, White, and other (includes selection for American Indian, Alaska Native, other, or unknown or declined to answer). Race and ethnicity were collected as part of cohort demographic characterization.

**Table 1.** Transition Probabilities and Utilities Used in Base Markov Decision Models

State	Estimate, per cycle	Source
Transition probabilities		
Symptomatic hernia to hernia incarceration	0.071	Marsman et al^13^
Successful incarcerated hernia reduction	0.333	Marsman et al,^13^ Choi et al^14^
Symptomatic hernia to flood syndrome (ruptured hernia)	0.008	Marsman et al.^13^
Resolved hernia to symptomatic hernia (hernia recurrence)	0.01	Ammar et al,^15^ Oh et al,^16^ Belli et al^17^
Probability of postoperative mortality, elective setting	pVPS_mortality^a^	Mahmud et al^9^
Probability of postoperative mortality, emergent setting	pVPS_eMortality^a^	Mahmud et al^9^
Probability of complicated postoperative recovery, elective setting	pVPS_comp^a^	Mahmud et al^11^
Probability of complicated postoperative recovery, emergent setting	pVPS_eComp^a^	Mahmud et al^11^
Probability of mortality attributable to baseline liver disease (ie, no surgery)	pMELDNa_mortality^a^	Derived in the present study
Base state utilities		
Resolved hernia (utility associated with cirrhosis alone, assume decompensated status)	0.324	Sherman et al,^18^ Siebert et al,^19^ Younossi et al,^20^ Chong et al,^21^ McPhail et al,^22^ Mclernon et al^23^
Symptomatic hernia (90% of utility associated with resolved hernia)	0.292	Poobalan et al,^24^ Stylopolous et al^25^
Transition state utilities (penalties)		
Incarceration (22.8% decrease from best health state)	−0.148	Bass et al,^26^ De Mestral et al^27^
Flood syndrome (42.9% decrease from best health state)	−0.278	Bass et al,^26^ De Mestral et al^27^
Elective surgery (8.8% decrease from best health state)	−0.057	Bass et al,^26^ De Mestral et al^27^
Emergent surgery (21.8% decrease from best health state)	−0.141	Bass et al,^26^ De Mestral et al^27^
Complicated surgical recovery (20.8% decrease from best health state)	−0.134	Bass et al,^26^ De Mestral et al^27^

Abbreviations: MELD-NA, model for end-stage liver disease–sodium; p, probability; VPS, VOCAL-Penn Score.

^a^ Varies by current MELD-Na in the model cycle. Further information is provided in eTable 1 in the Supplement.

### Statistical Analysis 

#### VOCAL-VASQIP Data

In the VOCAL-VASQIP cohort, patients were stratified by operative and nonoperative treatment, and data were summarized using medians and IQRs for continuous data and counts and percentages for categorical data. Comparisons were made using the Wilcoxon Rank-sum test for continuous data and χ^2^ tests for categorical data. To visualize differences in projected 180-day mortality with different operative scenarios, we plotted mean predicted probabilities of mortality across MELD-Na strata and fit linear regression models, given apparent linearity of data by visual inspection. Data were also plotted in reference to projected 180-day mortality from a logistic regression model fit with MELD-Na score alone (ie, an estimate of nonoperative short-term mortality). To visualize excess mortality attributable to surgical treatment, we plotted the difference between projected mortality from VPS-derived linear regression models and the logistic regression model with MELD-Na score alone.

#### Markov Decision Model

Using transition probabilities and utilities noted in Table 1 and eTable 1 in the Supplement, a series of base models were run, iterating over initial MELD-Na scores from 6 to 25 in 1-point increments. The time horizon for each model was 10 cycles (ie, approximately 5 years). A half-cycle correction was performed at Markov node entry and exit. To model progression of liver disease over time, the MELD-Na score increased by 0.75 points per cycle (derived from the mean of the mean MELD-Na increase in the VOCAL-VASQIP cohort per year: 1.59) (eFigure 2 in the Supplement). A final payoff was added corresponding to the projected median life expectancy for patients with the exit MELD-Na score, multiplied by the corresponding yearly utility of the final base state (eTable 2 in the Supplement). Estimates for median life expectancy were obtained from VOCAL-VASQIP patients who did not receive surgical treatment, and survival data were stratified by MELD-Na score to determine median survival in years. Smoothed estimates were obtained from post hoc projected median survival from a linear regression model fit with log-transformed MELD-Na (eFigure 3 in the Supplement).

The outcome of the Markov decision model was quality-adjusted life-years (QALYs). Expected QALYs for nonsurgical management vs surgical treatment were plotted across the range of initial MELD-Na scores to identify an equivalence decision threshold. Stacked bar graphs were used to visualize distributions of final base states for each approach. To visualize probability distributions of expected value for different management decisions in scenarios on either side of the decision threshold (±4 MELD-Na points), we performed 100 000 trial Monte Carlo simulations. The difference in QALYs between operative treatment and nonoperative management were plotted for each trial to visualize the proportion of patients for whom surgical treatment was the preferred strategy at each MELD-Na score.

#### Sensitivity Analyses

To identify model sensitivity to key transition probabilities, we constructed tornado diagrams for probabilities across ranges spanning 25% to 100% of the base values in either direction, depending on the variable. These conservative ranges were selected a priori based on expert opinion owing to lack of empirical literature. We also explored the impact of changing utility reductions associated with the symptomatic hernia state (expressed as percentage decrement from the resolved hernia state) in the tornado diagrams. These were performed at MELD-Na scores plus or minus 4 points on either side of the equivalence decision threshold identified in the primary analysis. Next, given the possibility that specific hernia characteristics and varying degrees of ascites (which are not readily ascertainable in the data set used to derive the transition probabilities) could impact the rate of postoperative complications, we performed a 2-way sensitivity analysis in which the probability of a complicated recovery was varied by a factor of plus or minus 50% across all ranges of initial MELD-Na score. Finally, to evaluate the impact of uncertainty in model inputs varying simultaneously, we performed probabilistic sensitivity analyses at the decision threshold and plus or minus 4 MELD-Na points in successive 100 000 trial Monte Carlo simulations. Transition probabilities and percentage utility decrement relative to the resolved hernia state were sampled from β distributions in which approximately 95% of the probability distribution covered the aforementioned probability ranges.^28^

*P* values were 2-sided, and statistical significance was set at α = .05. Analyses were performed using Stata statistical software version 17.0/BE (StataCorp) and TreeAge Pro Healthcare version 2022 (TreeAge Software). Data were analyzed from January 1 to May 1, 2022.

## Results

### VOCAL-VASQIP Abdominal Hernia Cohort

**Figure 2.**
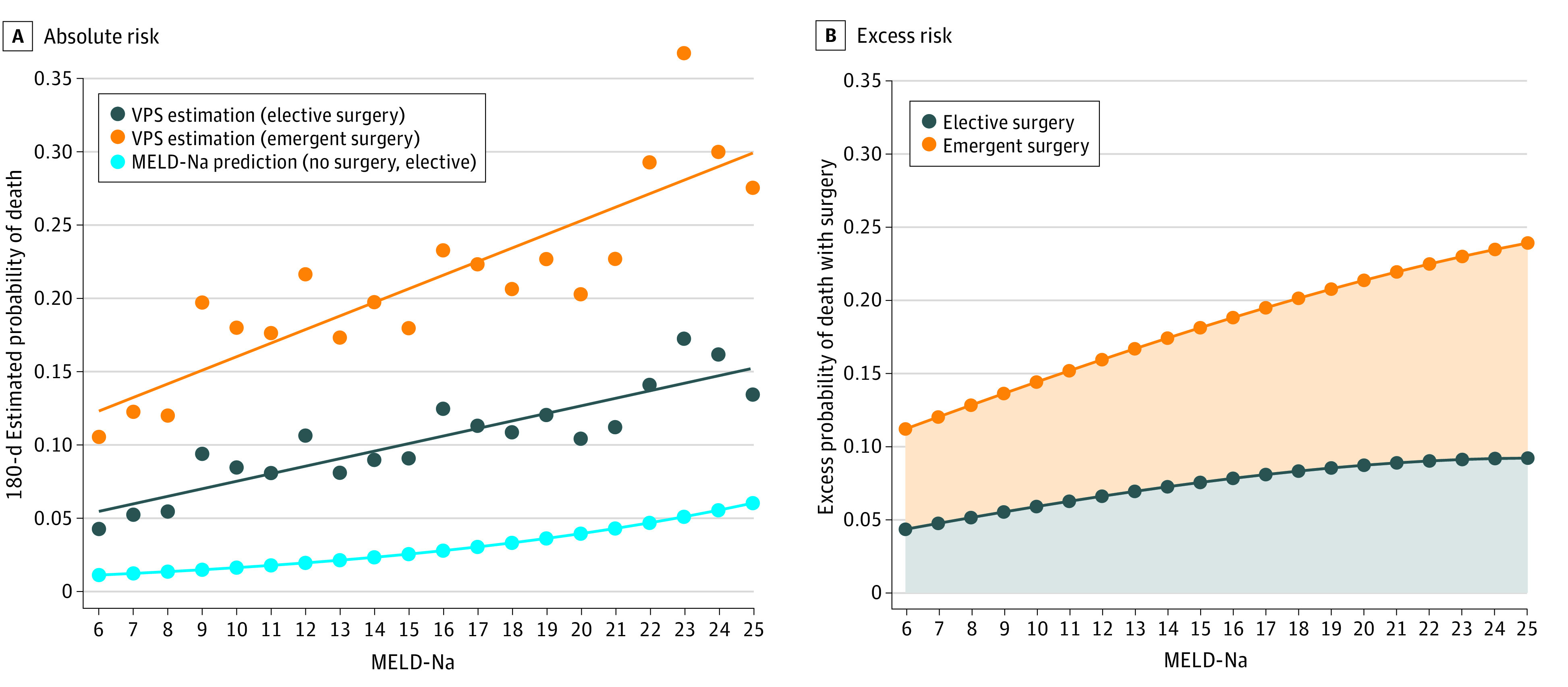
Projected Risk of 180-Day Mortality with Operative and Nonoperative Strategies Expressed MELD-Na indicates model for end-stage liver disease–sodium; VPS, VOCAL-Penn Score.

A total 2740 patients with cirrhosis (median [IQR] age, 62 [56-66] years; 2699 [98.5%] men) and a symptomatic abdominal hernia were identified. Of these, 1752 (63.9%) did not receive operative treatment within 1 year of surgical consultation, while 988 patients (36.1%) did receive surgical treatment. Patients who underwent surgical treatment, compared with those who did not, had lower body mass index (BMI; calculated as weight in kilograms divided by height in meters squared) (median [IQR], 26.8 [23.7-30.5] vs 27.4 [24.3-31.6]; *P* = .001), higher proportions of CTP class A cirrhosis (734 patients [74.3%] vs 1072 [61.2%]; *P* < .001), lower MELD-Na scores (median [IQR], 9 [6-13] points vs 11 [7-16] points; *P* < .001), higher albumin (median [IQR], 3.6 [3.1-4.1] g/dL vs 3.3 [2.8-3.8] g/dL [to convert to grams per liter, multiply by 10]; *P* < .001), higher platelet count (median [IQR, 142 [100-194]×10^3^/µL vs 121 [82-178] ×10^3^/µL [to convert to ×10^9^/L, multiply by 1]; *P* < .001), and were less likely to have major medical comorbidities (Table 2). There was no significant difference in the presence of ascites between the surgical treatment (533 patients [41.6%] and nonsurgical treatment (787 patients [44.9%]) groups (*P* = .09). Figure 2A displays estimated probabilities of 180-day mortality in nonoperative, elective operative, and emergency operative scenarios, as well as fitted linear regression lines. The estimated excess probabilities of death with surgical treatment are shown in Figure 2B. Detailed transition probability inputs derived from these models and probabilities of complicated surgical recovery estimated from VPS decompensation models are shown in eTable 1 in the Supplement.

**Table 2.** Characteristics of Abdominal Hernia Patients Who Did or Did Not Proceed to Surgical Treatment

Characteristic	Received surgical treatment, No. (%)		*P* value
	No (N = 1752)	Yes (N = 988)	
Age, median (IQR), y	61 (56-67)	62 (56-66)	.72
Sex			
Women	26 (1.5)	15 (1.5)	.94
Men	1726 (98.5)	973 (98.5)	
Race and ethnicity			
Asian	17 (1.0)	14 (1.4)	.69
Black	244 (13.9)	139 (14.1)	
Hispanic	135 (7.7)	80 (8.1)	
White	1205 (68.8)	681 (68.9)	
Other^a^	151 (8.6)	74 (7.5)	
Smoking			
Never	545 (31.6)	276 (28.2)	.15
Former	615 (35.6)	356 (36.3)	
Current	566 (32.8)	348 (35.5)	
Alcohol use disorder	403 (23.0)	210 (21.3)	.29
BMI, median (IQR)	27.4 (24.3-31.6)	26.8 (23.7-30.5)	.001
Etiology of liver disease			
HCV	242 (13.8)	187 (18.9)	.003
Hepatitis B virus	14 (0.8)	6 (0.6)	
Alcohol-related liver disease	726 (41.5)	401 (40.6)	
HCV and ALD	422 (24.1)	242 (24.5)	
Non-alcoholic fatty liver disease	291 (16.6)	129 (13.1)	
Other	54 (3.1)	22 (2.2)	
CTP class			
A	1072 (61.2)	734 (74.3)	<.001
B	651 (37.2)	241 (24.4)	
C	29 (1.7)	13 (1.3)	
MELD, median (IQR)	9 (6-14)	6 (6-9)	<.001
MELD-Na, median (IQR)	11 (7-16)	9 (6-13)	<.001
Decompensated cirrhosis	1089 (62.2)	533 (53.9)	<.001
Ascites	787 (44.9)	411 (41.6)	.09
Obesity	561 (32.0)	282 (28.5)	.06
Diabetes	946 (54.0)	487 (49.3)	.02
Coronary artery disease	436 (24.9)	203 (20.5)	.01
Heart failure	292 (16.7)	130 (13.2)	.02
Atrial fibrillation	209 (11.9)	101 (10.2)	.18
CKD	636 (38.3)	299 (31.9)	.001
COPD	714 (43.0)	407 (43.5)	.81
Laboratory measurements, median (IQR)			
Sodium, mEq/L	138 (135-140)	138 (136-140)	.007
Creatinine, mg/dL	0.9 (0.8-1.2)	0.9 (0.8-1.1)	.43
Albumin, g/dL	3.3 (2.8-3.8)	3.6 (3.1-4.1)	<.001
Total bilirubin, mg/dL	1.1 (0.7-1.9)	0.9 (0.6-1.6)	<.001
Platelet Count, ×10^3^/µL	121 (82-178)	142 (100-194)	<.001
INR	1.2 (1.1-1.5)	1.2 (1.1-1.3)	<.001

Abbreviations: ALD, alcohol-related liver disease; BMI, body mass index (calculated as weight in kilograms divided by height in meters squared); CTP, Child-Turcotte-Pugh; HCV, hepatitis C virus; MELD, model for end-stage liver disease; INR, international normalized ratio; MELD-Na, model for end-stage liver disease–sodium.

SI conversion factors: To convert albumin to grams per liter, multiply by 10; bilirubin to micromoles per liter, multiply by 17.104; creatinine to micromoles per liter, multiply by 76.25; platelet count to ×10^9^/L, multiply by 1.

^a^ Other race or ethnicity includes selection for American Indian, Alaska Native, other, or unknown or declined to answer.

### Primary Markov Decision Model Results

**Figure 3.**
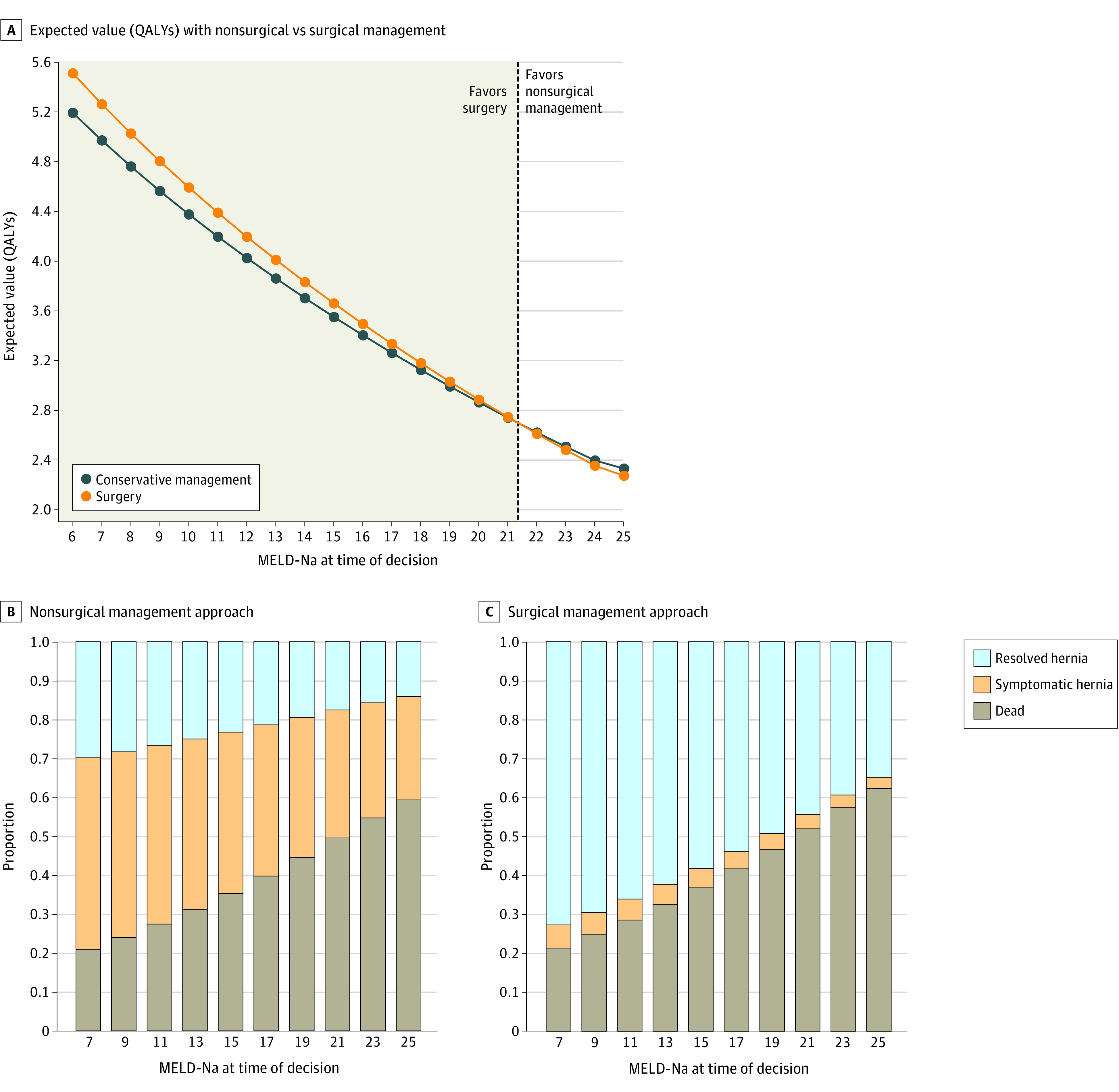
Results of Primary Markov Decision Analysis MELD-Na indicates model for end-stage liver disease–sodium; QALY, quality-adjusted life-years.

Iterating the model over MELD-Na scores at the time of initial decision (surgical treatment vs nonsurgical management), a decision threshold was identified at a MELD-Na score of 21.3 points, at which point expected QALYs were the same with surgical and nonsurgical management (Figure 3A). For MELD-Na scores less than 21.3 points, surgical treatment was the preferred strategy, whereas nonsurgical management was favored for MELD-Na scores greater than 21.3 points. The distribution of health states at the conclusion of the Markov processes with each management strategy are shown in Figure 3B and C. Although the proportion of patients experiencing death was higher for all initial MELD-Na values with a surgical treatment decision (MELD-Na score of 7 points: surgical treatment: 21.6% of patients who received surgical treatment vs 21.2% of patients who received nonsurgical management; MELD-Na score of 25 points: 62.4% of patients who received surgical treatment vs 59.5% of patients who received nonsurgical management), the higher expected QALYs observed in patients with MELD-Na scores less than 21.3 points was driven primarily by accumulated time in the resolved hernia state. In Monte Carlo simulations performed at initial MELD-Na scores of 17 or 25 points, a large proportion of patients who received surgical treatment had higher expected QALYs than nearly all patients in the nonsurgical management strategy. However, the mean expected QALYs with initial MELD-Na score of 25 were reduced in the surgical treatment strategy primarily owing to an increased short-term mortality associated with the operation (eFigure 4 in the Supplement). In plots of the difference in expected QALYs, 68.1% (95% CI, 67.8%-68.4%) of patients with a MELD-Na score of 17 points and 54.2% (95% CI, 54.0%-54.6%) of patients with a MELD-Na score of 25 points would have higher expected QALYs with surgical treatment (eFigure 4 in the Supplement).

### Sensitivity Analysis

In 1-way sensitivity analyses at MELD-Na scores of 17 and 25 points, the model was sensitive to 3 variables: hernia incarceration probability, postoperative hernia recurrence probability, and the proportional utility decrement associated with the symptomatic hernia state. To evaluate the impact of these variables on primary results, we performed serial 3-way sensitivity analyses. With varying probabilities of hernia recurrence and symptomatic hernia utility decrement across baseline MELD-Na scores, the decision threshold to favor nonoperative management shifted to a lower MELD-Na score for higher recurrence probabilities and lower utility decrements (eFigure 5 in the Supplement). For example, a patient with an 8% utility decrement (ie, less symptomatic hernia) and 2% 6-month probability of hernia recurrence would favor surgical treatment for MELD-Na scores less than 17 points, whereas a patient with a 12% utility decrement and a 1% 6-month probability of hernia recurrence would favor surgical treatment for MELD-Na scores less than 24 points. As expected, as the 6-month probability of hernia incarceration increases, initial surgical treatment would be favored for progressively higher MELD-Na scores (eFigure 5 in the Supplement). In a sensitivity analysis varying the probability of complicated recovery in both emergent and elective settings, there was minimal movement in the decision threshold to favor nonoperative management vs surgical treatment, remaining between MELD-Na scores of 21 and 22 points (eFigure 6 in the Supplement). Finally, the primary results were found to be robust in probabilistic sensitivity analyses: surgical treatment was favored in 96.9% (95% CI, 96.8%-97.0%) of trials at a MELD-Na score of 17 points, 57.1% (95% CI, 56.8%-57.4%) of trials at a MELD-Na score of 21 points, and 1.7% (95% CI, 1.6%-1.8%) of trials at a MELD-Na score of 25 points (eFigure 7 in the Supplement).

## Discussion

In this decision-analytic Markov study, we identified clinical thresholds based on MELD-Na scores to inform surgical decision-making for patients with cirrhosis with symptomatic abdominal hernias. Our results suggest that surgical treatment would generally be favored in patients with MELD-Na scores less than 21.3 points. Model results were sensitive to varying probabilities of postoperative hernia recurrence and hernia incarceration, as well as the utility decrement associated with the symptomatic hernia state.

A major impetus for exploring cirrhosis surgical risk in detail is the recognition that historic estimation models, such as the MRS, overestimate risk for many patients.^9^ This contributes to a culture of risk aversion in which patients with cirrhosis are denied elective surgical treatment based on a perception of prohibitive risk. A 2022 study by Johnson et al^29^ reported that a substantial proportion ( approximately 30%) of patients with cirrhosis who underwent emergency hernia repair could have safely received elective surgical treatment in the previous year. Our data build on these findings by explicitly exploring decision thresholds at which surgical treatment is expected to maximize QALYs relative to nonsurgical management in the elective setting.

A unique feature of our study is the inclusion of data from a cohort of patients with symptomatic hernias who never received surgical treatment, a subgroup that has not been previously included in comprehensive modeling efforts, to our knowledge. Similar to the findings of Johnson et al,^29^ our results support the hypothesis of status quo risk aversion in cirrhosis surgical decision-making. Most patients (88%) in the nonoperative cohort had MELD-Na scores less than 21 points at the time of surgical referral, suggesting that many could have received surgical treatment with reasonable expectation of increased QALYs. These results imply that currently perceived thresholds for acceptable surgical risk are lower than estimated by our decision model and should justifiably be shifted toward higher MELD-Na values. Risk averse behavior by clinicians and patients is not surprising, as fear of the unknown has been demonstrated to influence decision-making in other clinical scenarios.^30,31,32^ Models, such as those presented here, can help clinicians and patients to make more informed decisions.

In sensitivity analyses, the MELD-Na decision threshold was sensitive to 3 key parameters: the decrement in utility associated with a symptomatic hernia, the probability of postoperative hernia recurrence, and the probability of hernia incarceration. This highlights several important issues. First, because the estimated mortality rate was higher with surgical treatment regardless of MELD-Na score, the patient’s perspective and reported symptoms are critical in helping identify a reasonable threshold to recommend surgical treatment over nonsurgical management. For a patient with less severe or fewer symptoms, the threshold for surgical treatment may be substantially higher than that for a patient with more or more severe symptoms, with an approximate MELD-Na score range of 17 to 25 points in the sensitivity ranges of this study. This emphasizes the importance of shared decision-making for this clinical scenario, and future dedicated tools may assist with this. Second, the model sensitivity to this utility suggests future areas for research, in particular exploration of patient-reported utilities of various states associated with abdominal hernias. To our knowledge, there are no data on this subject; therefore, several assumptions had to be made in models, such as the decrement in utility resulting from a symptomatic hernia. Third, the sensitivity of the models based on probability of hernia recurrence underscores the importance of surgeon experience. Ideally, patients should be referred to high-volume liver transplantation centers with surgeons and anesthesiologists accustomed to caring for patients with cirrhosis and with expertise in managing postoperative complications and decompensation. Finally, the sensitivity of results to the probability of hernia incarceration highlights an important knowledge gap regarding the natural history of abdominal hernias in patients with cirrhosis. Indeed, very limited data exist to estimate this probability, and the risk of incarceration is expected to vary based on characteristics of the hernia and patient (eg, by location, size, presence or absence of ascites).

We acknowledge there are additional hernia-related considerations that may influence perceived risk of postoperative complications and the decision to pursue surgical treatment. These include hernia location, aperture of the fascial defect, degree of involvement of fat or bowel in the hernia, among other features. Severity of ascites may also impact the risk of postoperative complications. Given the paucity of literature on the natural history of hernias in patients with cirrhosis, this decision tool must be interpreted in this context. However, in a final sensitivity analysis, we explored the impact of varying probabilities of complicated surgical recovery from hernia repair and found no significant change in the decision threshold to favor surgical treatment vs nonsurgical management. This is consistent with single-center prospective studies in which even patients with primarily CTP class B or C cirrhosis and ascites could undergo elective hernia repair with acceptable risk.^33^

### Limitations

This study has important limitations. First, given the paucity of data on progression of abdominal hernias in patients with cirrhosis and quality of life associated with various states, key assumptions had to be made in Markov models. Although we used pre hoc data wherever possible, we addressed this issue through sensitivity analyses. Second, owing to lack of data granularity, we did not include the possibility of liver transplantation in this analysis; however, one can speculate regarding the possible impact of transplant candidacy. In patients with low MELD-Na scores, the threshold for surgical treatment may be lower if transplant is available as a rescue option. By contrast, patients with high MELD-Na scores who are on waiting lists and may receive a transplant offer in the short term may be better served by hernia repair at the time of transplantation. Future studies may address this possibility in detail. Third, we could not explicitly explore the impacts of surgeon expertise and volume on projected outcomes, although this may be indirectly reflected in the 3-way sensitivity analyses. Fourth, we did not evaluate the possibility that patients experiencing postoperative decompensation could have long-term reductions in utility in the resolved hernia state. This is a limitation of the basic Markov model structure, which does not have memory beyond the previous state, although this could be evaluated in future studies with higher-order Markov chain models.^34^ Fifth, to estimate probabilities of complicated postoperative course we used the VPS for decompensation. While these likely correlate well with a complex postoperative course, complications that reduce postoperative utility unrelated to cirrhosis decompensation may not be captured. However, the impact of this is likely minimal, given that sensitivity analyses demonstrated minimal change to the threshold MELD-Na scores with changing probabilities of complicated postoperative courses.

## Conclusions

In this decision analytical model study, we demonstrate a novel approach to assessing surgical risk vs the risk of not operating in patients with cirrhosis with symptomatic abdominal hernias. Our model suggests that elective surgical treatment would maximize QALYs for most patients with MELD-Na less than 21.3 points, indicating that many patients do not receive surgical treatment when it may be favorable. Sensitivity of models to utilities associated with the symptomatic hernia state and probability of hernia recurrence emphasize the importance of the severity of patient-reported symptoms and surgeon and center experience in final decision-making. Future prospective research in patients with cirrhosis and abdominal hernias may help further clarify optimal decision thresholds for diverse patient scenarios.

## Notes

Supplement.
eMethods.
**eTable 1.** Transition Probabilities for Nonoperative Mortality, Postoperative Mortality, and Complicated Surgical Recovery, Stratified by MELD-Na and Elective vs Emergent Surgical Scenario **eTable 2.** Final Payoffs for Patients Surviving Through Final Markov Cycle **eFigure 1.** Complete Decision Tree with Markov Nodes **eFigure 2.** Distribution of Mean Change in MELD-Na Per Year in the Analytic Cohort **eFigure 3.** Estimates of Median Survival by MELD-Na to Inform Payoff Assigned After Last Markov Cycle **eFigure 4.** Expected Value Probability Distributions for Nonsurgical vs Surgical Management Strategies From Monte Carlo Simulations **eFigure 5.** 3-Way Sensitivity Analyses Including Probabilities of Postoperative Hernia Recurrence, Hernia Incarceration, and Utility Decrement Associated With the Symptomatic Hernia State **eFigure 6.** 2-Way Sensitivity Analysis with Varying Percentage Change in Probability of Complicated Surgical Recovery **eFigure 7.** Probabilistic Sensitivity Analyses Using Monte Carlo Simulations
