# Supplementary material for: Modeling Optimal Clinical Thresholds for Elective Abdominal Hernia Repair in Patients With Cirrhosis
Source: JAMA Netw Open. 2022 Sep 13;5(9):e2231601. doi: 10.1001/jamanetworkopen.2022.31601 (PMC9471978; doi:10.1001/jamanetworkopen.2022.31601)
Supplement: Supplement. — eMethods. eTable 1. Transition Probabilities for Nonoperative Mortality, Postoperative Mortality, and Complicated Surgical Recovery, Stratified by MELD-Na and Elective vs Emergent Surgical Scenario eTable 2. Final Payoffs for Patients Surviving Through Final Markov Cycle eFigure 1. Complete Decision Tree with Markov Nodes eFigure 2. Distribution of Mean Change in MELD-Na Per Year in the Analytic Cohort eFigure 3. Estimates of Median Survival by MELD-Na to Inform Payoff Assigned After Last Markov Cycle eFigure 4. Expected Value Probability Distributions for Nonsurgical vs Surgical Management Strategies From Monte Carlo Simulations eFigure 5. 3-Way Sensitivity Analyses Including Probabilities of Postoperative Hernia Recurrence, Hernia Incarceration, and Utility Decrement Associated With the Symptomatic Hernia State eFigure 6. 2-Way Sensitivity Analysis with Varying Percentage Change in Probability of Complicated Surgical Recovery eFigure 7. Probabilistic Sensitivity Analyses Using Monte Carlo Simulations [file jamanetwopen-e2231601-s001.pdf]

Supplemental Online Content

Mahmud N, Goldberg DS, Abu-Gazala S, Lewis JD, Kaplan DE. Modeling optimal clinical thresholds for elective abdominal hernia repair in patients with cirrhosis. *JAMA Netw Open*. 2022;5(9):e2231601. doi:10.1001/jamanetworkopen.2022.31601

eMethods.
eTable 1. Transition Probabilities for Nonoperative Mortality, Postoperative Mortality, and Complicated Surgical Recovery, Stratified by MELD-Na and Elective vs Emergent Surgical Scenario
eTable 2. Final Payoffs for Patients Surviving Through Final Markov Cycle
eFigure 1. Complete Decision Tree with Markov Nodes
eFigure 2. Distribution of Mean Change in MELD-Na Per Year in the Analytic Cohort
eFigure 3. Estimates of Median Survival by MELD-Na to Inform Payoff Assigned After Last Markov Cycle
eFigure 4. Expected Value Probability Distributions for Nonsurgical vs Surgical Management Strategies From Monte Carlo Simulations
eFigure 5. 3-Way Sensitivity Analyses Including Probabilities of Postoperative Hernia Recurrence, Hernia Incarceration, and Utility Decrement Associated With the Symptomatic Hernia State
eFigure 6. 2-Way Sensitivity Analysis with Varying Percentage Change in Probability of Complicated Surgical Recovery
eFigure 7. Probabilistic Sensitivity Analyses Using Monte Carlo Simulations

This supplemental material has been provided by the authors to give readers additional information about their work.

© 2022 Mahmud N et al. *JAMA Network Open.*

eMethods.

Estimation of Transition Probabilities Leading to Death

Key transition probabilities include the 180-day probability of death from symptomatic abdominal hernia, elective surgery, and emergent surgery states. The VOCAL-Penn Score (VPS) provides validated estimates of 180-day post-operative mortality for elective and emergent surgical scenarios,[9] however the probability of 180-day mortality in patients with symptomatic abdominal hernias managed non-operatively must be estimated from patients diagnosed with this condition *but who did not receive surgery*. To identify a group of such patients we utilized the Veterans Outcomes and Costs Associated with Liver Disease (VOCAL) cohort, which is a well-established longitudinal cohort of patients with cirrhosis in the Veterans Health Administration (VHA) identified between 1/1/2008 and 12/31/2018 using a VHA-validated algorithm.[13-15] Our group has previously merged the granular clinical data in VOCAL with manually-adjudicated surgical data from the Veterans Affairs Surgical Quality Improvement Program (VASQIP).[9] From this merged dataset we identified all patients with cirrhosis who were ever referred to outpatient surgery clinic for evaluation of an abdominal hernia in the VHA consult tables. All VHA consults require text descriptions for the referral reason; structured query language (SQL) queries were therefore used to identify abdominal hernia referrals. The referral text of each consult was manually reviewed for consistency. In this cohort, Current Procedure Terminology (CPT) codes were used to identify all patients who received surgical hernia repair within one year of surgery consultation, thus forming sub-cohorts of patients who received operative and non-operative management. For each patient we obtained detailed demographic (age, sex, race), body mass index (BMI), smoking history, alcohol use disorder (per the Alcohol Use Disorders Identification Test [AUDIT-C] instrument), and comorbidity data (diabetes, coronary artery disease, heart failure, atrial fibrillation, chronic kidney disease, chronic obstructive pulmonary disease), and laboratory data (sodium, creatinine, albumin, total bilirubin, platelet count, international normalized ratio), similar to prior methods.[16-19] Etiology of liver disease, Child-Turcotte-Pugh class, and prior cirrhosis decompensation were each determined using well-validated VHA algorithms.[13, 20, 21] All data were time-updated at the time of surgery referral; laboratory data were obtained in a 90-day window prior to referral, using the most recent values available. Model for end-stage liver disease (MELD) and MELD-sodium (MELD-Na) were computed from laboratory data. Mortality at 180 days from surgery referral was determined using the Vital Status file, which is well-validated in the VHA dataset.[22]

To estimate the probability of 180-day mortality in patients who did not receive hernia repair, we began with a logistic regression modeling approach. We performed univariable analyses to evaluate for non-linearity between continuous exposures and the outcome, and transformed variables using restricted cubic splines where appropriate. We then used reverse stepwise modeling including all possible exposures to identify a candidate model. Multiple clinician-driven variant models were then created, where *a priori* variables felt to be potentially clinically meaningful were reintroduced. A final model was selected based on minimization of the Bayesian Information Criterion (BIC). In the course of this modeling process, a univariable model fit with MELD-Na alone was found to minimize BIC and yield adequate discrimination for the outcome (C-statistic > 0.7). As such, we chose to focus subsequent modeling efforts, including in the decision-analytic Markov model, based on MELD-Na score. This approach has the additional benefit of clinical familiarity given that MELD-Na is the most commonly used prediction score in the field of chronic liver disease. Predicted 180-day mortality with non-operative management from this regression model, stratified by MELD-Na, are shown in **Supplemental Table 1**.

Transition probabilities for post-operative death were calculated using the VPS in elective and emergency scenarios, stratified by MELD-Na, and fit with a least squares regression model (**Table 1**, **Supplemental Table 1**).[9] A similar approach was used to estimate the probability of complicated post-operative recovery using the VPS score for post-operative decompensation (**Table 1**, **Supplemental Table 1**).[11] Of note, given that an emergent surgical scenario such as hernia incarceration or rupture would likely entail worsened

© 2022 Mahmud N et al. *JAMA Network Open.*

laboratory values, we assumed a 10% increase in total bilirubin and a 10% decrease in albumin for associated calculations using the VPS models.

Estimation of Remaining Transition Probabilities and Utilities

Prior studies from Marsman, et al. and Choi, et al. were used to estimate six-month probabilities of hernia incarceration, successful hernia reduction, and hernia rupture (Flood syndrome; **Table 1**).[23, 24] The probability of post-operative hernia recurrence in patients with cirrhosis was estimated using data from Ammar, et al., Oh, et al., and Belli, et al.[25-27] As noted above, the remaining transition probabilities were estimated from models using VOCAL-VASQIP cohort data, with escalating probabilities with increasing MELD-Na (**Table 1**, **Supplemental Table 1**). Regarding model utilities, the resolved hernia state utility was estimated from several studies assessing utilities associated with decompensated cirrhosis.[28-33] The symptomatic hernia state was assumed to have 90% of the utility associated with the resolved hernia state, adapted from studies by Poobalan et al., Stylopolous et al.[34, 35] Finally, given that no studies could be found describing utilities associated with various hernia complications and immediate post-operative hernia repair states, we approximated utility decrements from comparable surgical scenarios in studies by Bass, et al. and De Mestral, et al. (summarized in **Table 1**).[36, 37]

© 2022 Mahmud N et al. *JAMA Network Open.*

eTable 1. Transition Probabilities for Nonoperative Mortality, Postoperative Mortality, and Complicated Surgical Recovery, Stratified by MELD-Na and Elective vs Emergent Surgical Scenario*

MELD-Na	**pMELDNa_mortality** (180-day mortality with non-operative management)	**pVPS_mortality** (180-day mortality with elective surgery)	**pVPS_eMortality** (180-day mortality with emergent surgery)	**pVPS_comp** (probability of complicated post-operative course, elective setting)	**pVPS_eComp** (probability of complicated post-operative course, emergent setting)
6	0.012	0.056	0.124	0.083	0.176
7	0.013	0.061	0.133	0.090	0.185
8	0.015	0.066	0.142	0.098	0.194
9	0.016	0.071	0.152	0.105	0.204
10	0.017	0.076	0.161	0.113	0.213
11	0.019	0.081	0.170	0.120	0.223
12	0.020	0.086	0.179	0.128	0.232
13	0.022	0.091	0.189	0.135	0.242
14	0.024	0.097	0.198	0.143	0.251
15	0.026	0.102	0.207	0.150	0.261
16	0.029	0.107	0.216	0.158	0.270
17	0.031	0.112	0.226	0.165	0.280
18	0.034	0.117	0.235	0.173	0.289
19	0.037	0.122	0.244	0.180	0.298
20	0.040	0.127	0.253	0.188	0.308
21	0.044	0.132	0.263	0.195	0.317
22	0.048	0.138	0.272	0.203	0.327
23	0.052	0.143	0.281	0.210	0.336
24	0.056	0.148	0.290	0.218	0.346
25	0.061	0.153	0.300	0.225	0.355

** Variable names in columns correspond to transition probabilities noted in **Table 1***

© 2022 Mahmud N et al. *JAMA Network Open.*

eTable 2. Final Payoffs for Patients Surviving Through Final Markov Cycle†

Final MELD-Na	Life-Years*	Final Payoff in Resolved Hernia State (QALYs)	Final Payoff in Symptomatic Hernia State (QALYs)
6	8.601	5.573	5.016
7	7.934	5.141	4.627
8	7.356	4.767	4.290
9	6.846	4.436	3.993
10	6.390	4.141	3.727
11	5.978	3.874	3.486
12	5.602	3.630	3.267
13	5.255	3.405	3.065
14	4.935	3.198	2.878
15	4.636	3.004	2.704
16	4.357	2.823	2.541
17	4.095	2.654	2.388
18	3.847	2.493	2.244
19	3.613	2.341	2.107
20	3.391	2.197	1.978
21	3.180	2.061	1.855
22	2.979	1.930	1.737
23	2.787	1.806	1.625
24	2.603	1.687	1.518
25	2.426	1.572	1.415
26	2.256	1.462	1.316
27	2.093	1.356	1.221
28	1.936	1.255	1.129
29	1.784	1.156	1.040
30	1.637	1.061	0.955
31	1.495	0.969	0.872

** Estimates derived from log-linear regression model in **Supplemental Figure 3***
† All final QALY payoffs in table discounted by utility of final state (e.g., symptomatic hernia or resolved hernia)

© 2022 Mahmud N et al. *JAMA Network Open.*

eFigure 1. Complete Decision Tree with Markov Nodes

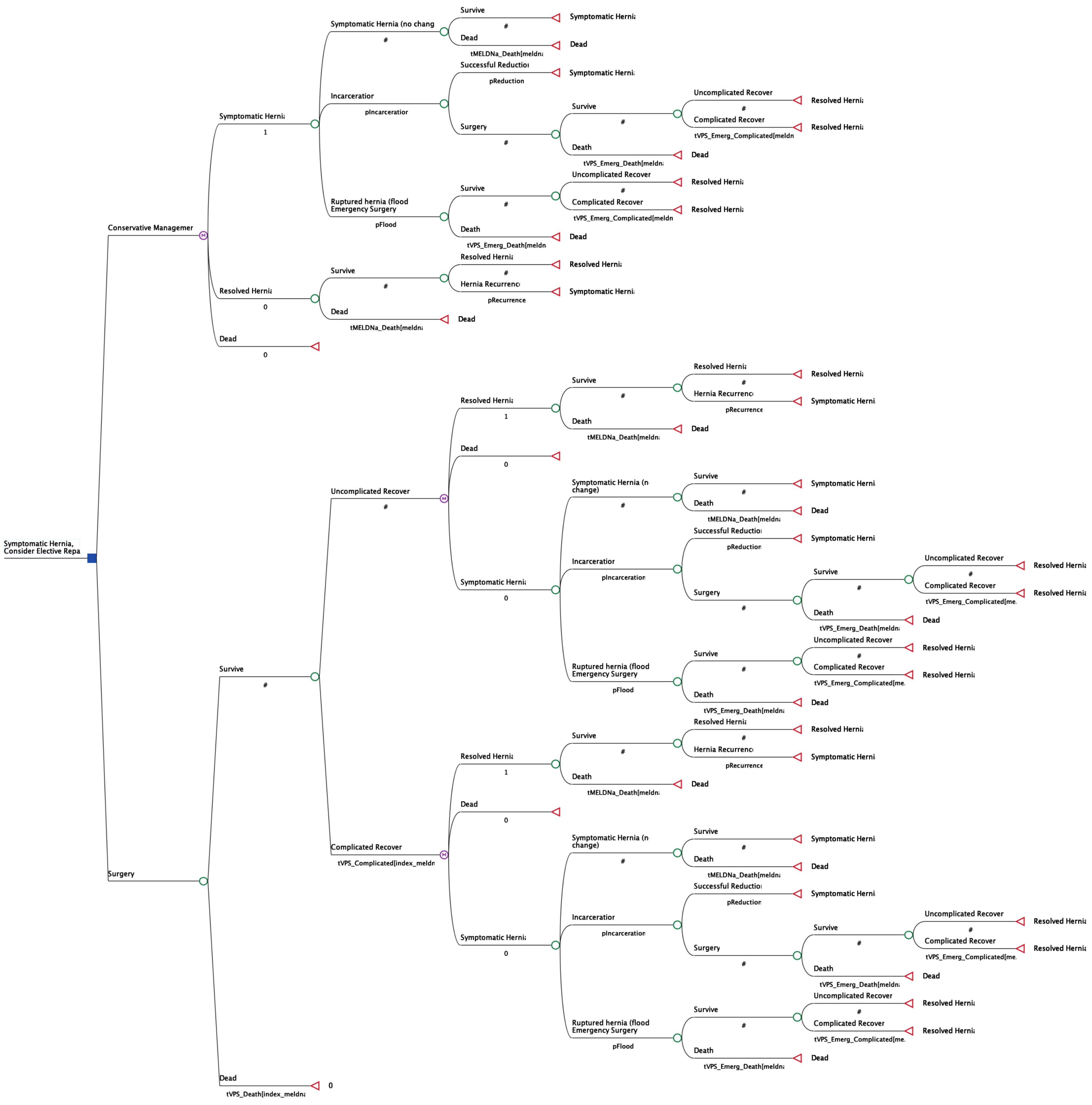

© 2022 Mahmud N et al. *JAMA Network Open.*

eFigure 2. Distribution of Mean Change in MELD-Na Per Year in the Analytic Cohort

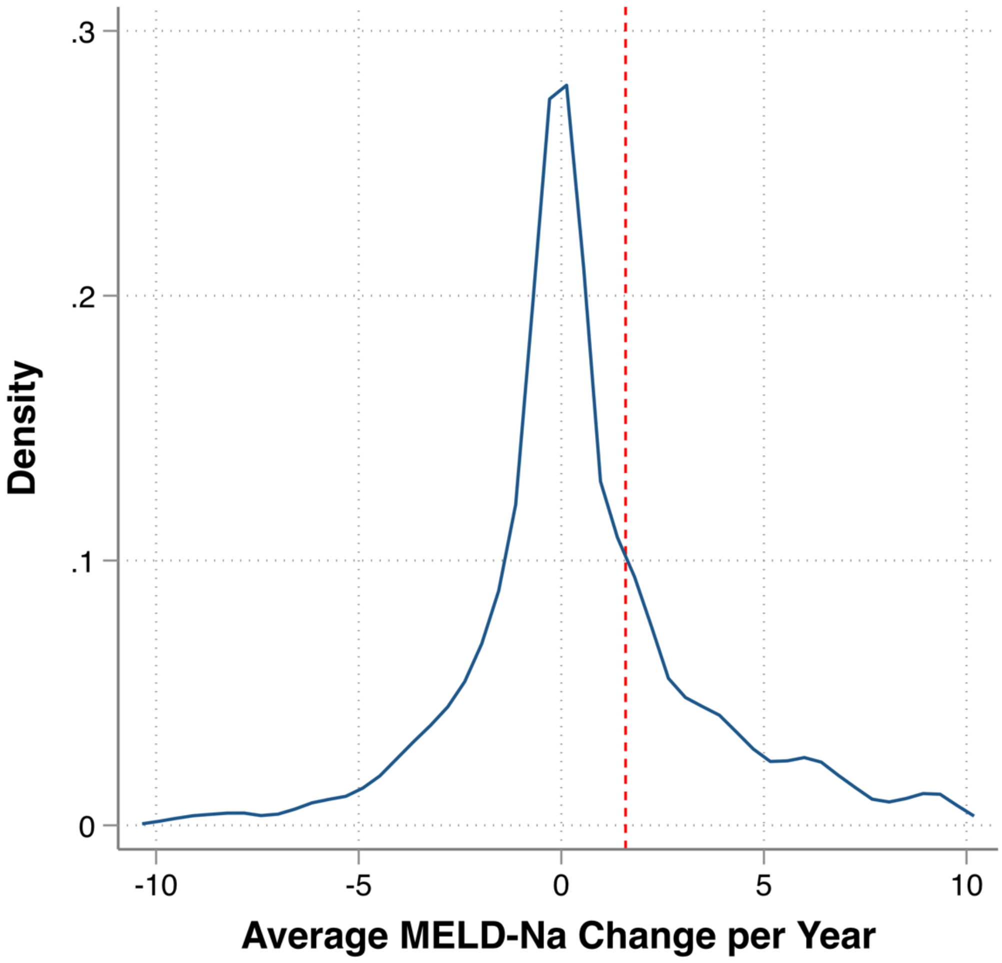

** Vertical dashed line corresponds to average change in annual MELD-Na: 1.53*

© 2022 Mahmud N et al. *JAMA Network Open.*

eFigure 3. Estimates of Median Survival by MELD-Na to Inform Payoff Assigned After Last Markov Cycle

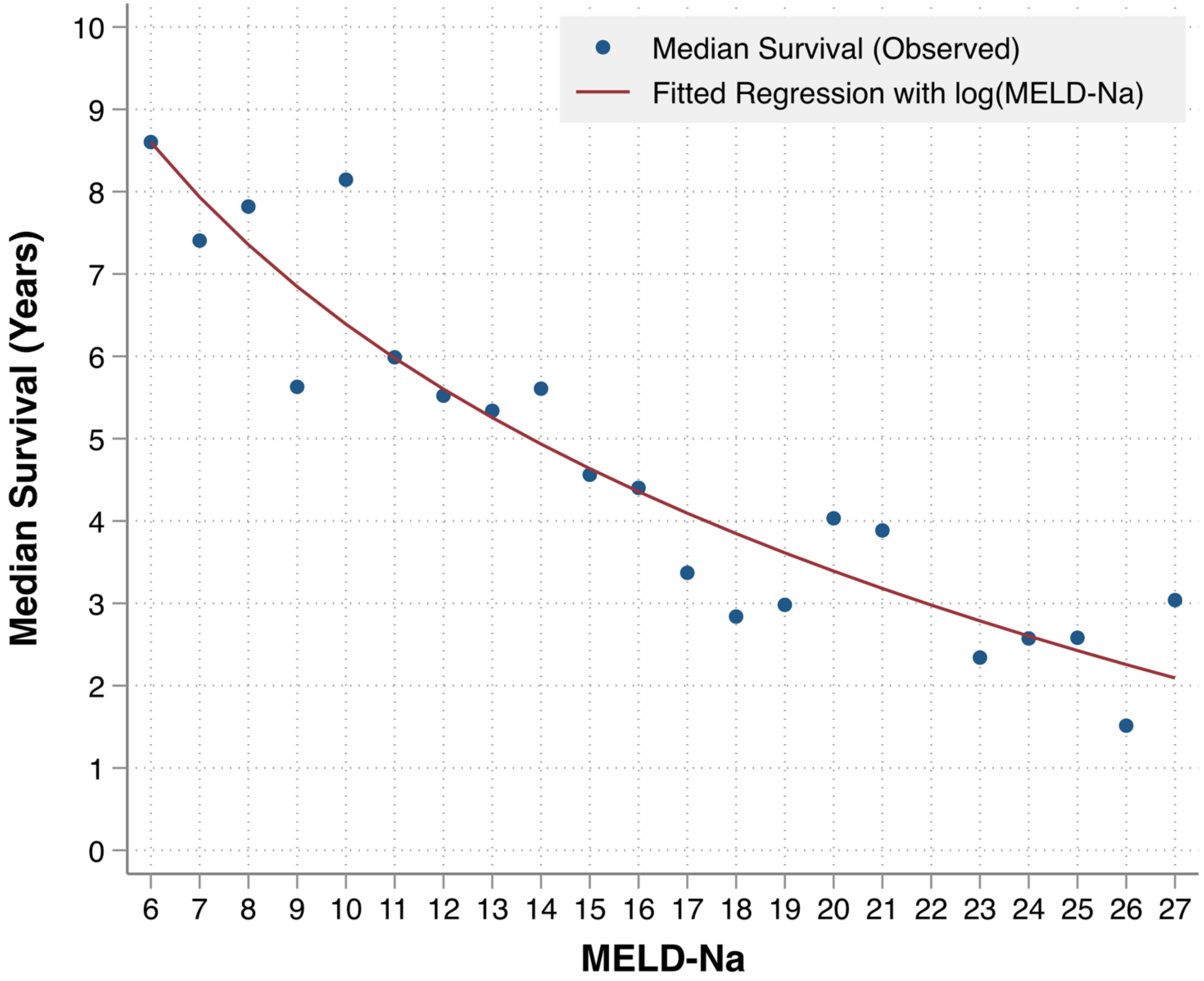

**Note that MELD-Na 22 datapoint was excluded as an outlier*

© 2022 Mahmud N et al. *JAMA Network Open.*

eFigure 4. Expected Value Probability Distributions for Nonsurgical vs Surgical Management Strategies From Monte Carlo Simulations

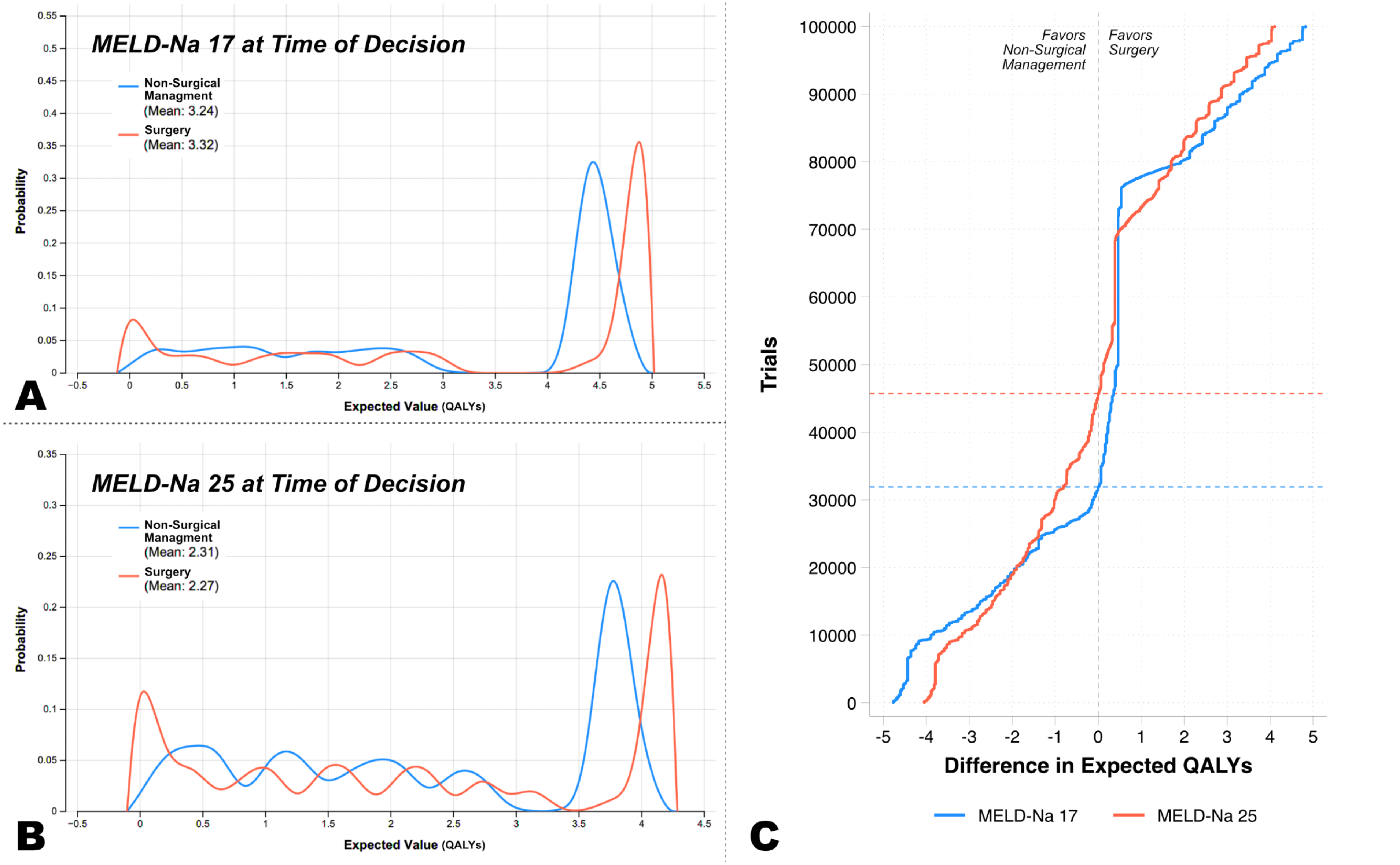

** In panel C, horizontal dashed lines correspond to trials where there is no difference in expected QALYs between surgery and non-surgical management, for both MELD-Na 17 and MELD-Na 25*

© 2022 Mahmud N et al. *JAMA Network Open.*

eFigure 5. 3-Way Sensitivity Analyses Including Probabilities of Postoperative Hernia Recurrence, Hernia Incarceration, and Utility Decrement Associated With the Symptomatic Hernia State*

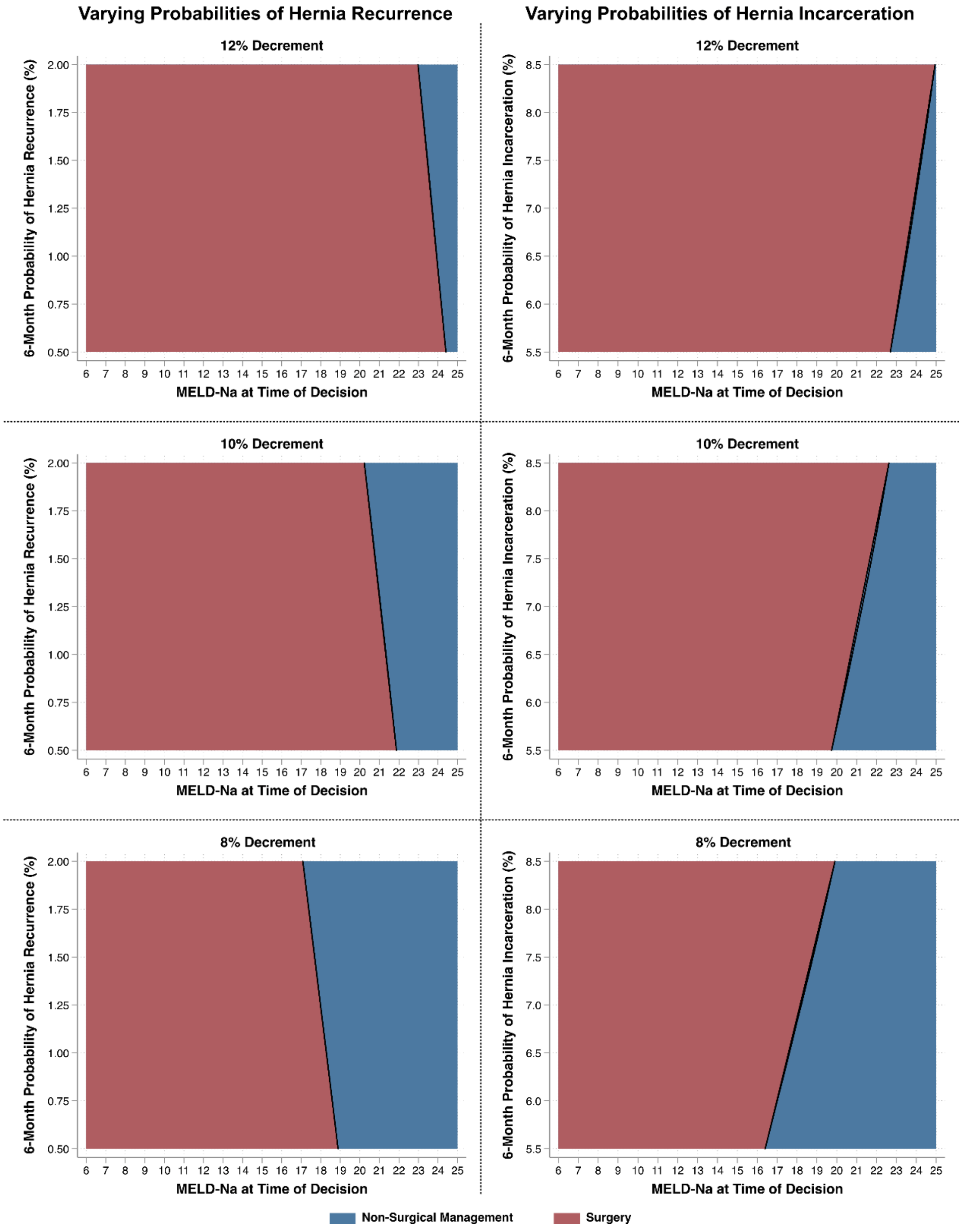

** Decrement refers to the percentage by which the symptomatic hernia state utility is reduced related to the resolved hernia state utility*

© 2022 Mahmud N et al. *JAMA Network Open.*

eFigure 6. 2-Way Sensitivity Analysis with Varying Percentage Change in Probability of Complicated Surgical Recovery

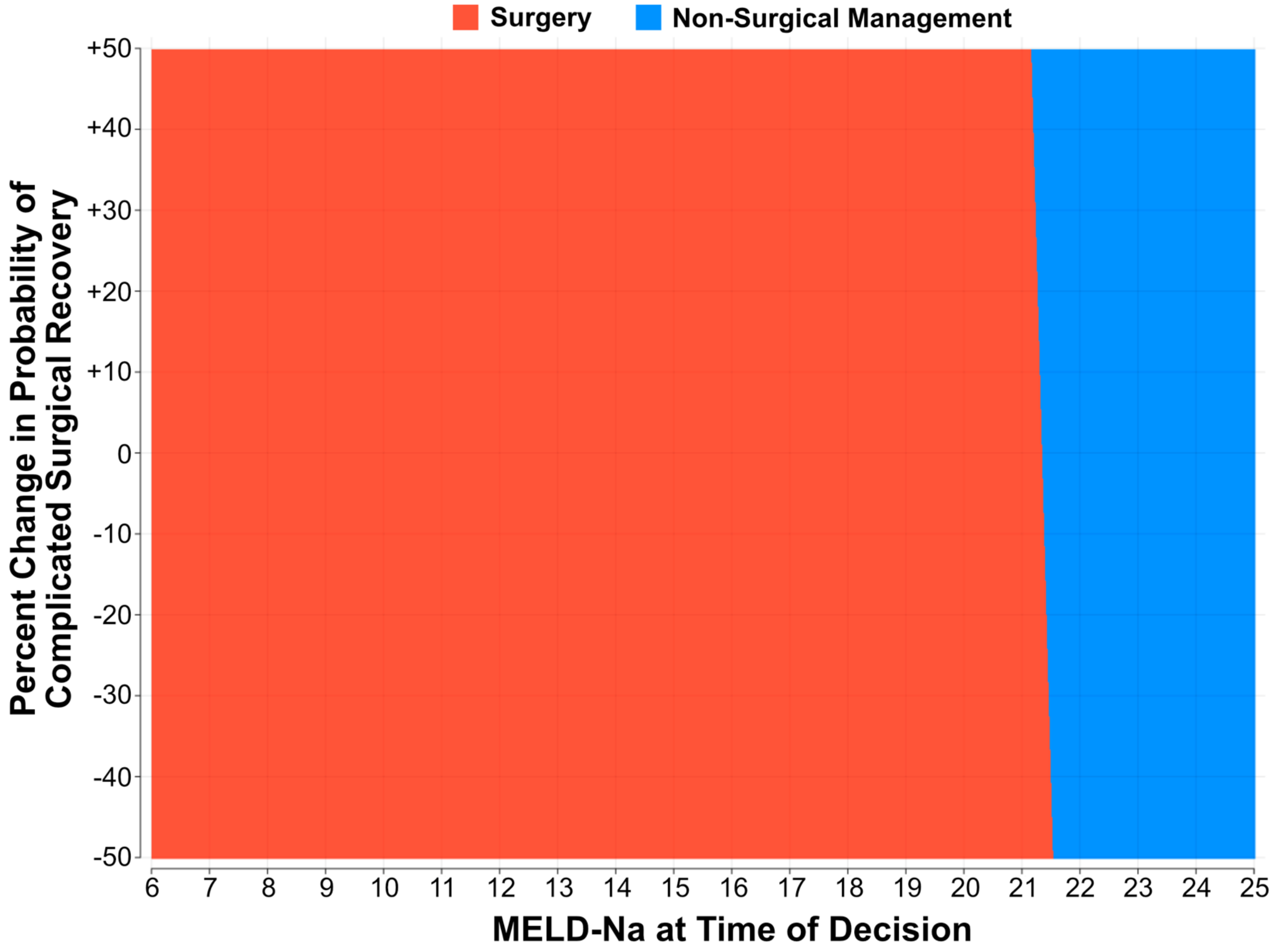

** Note that percent change (y-axis) refers to the factor by which the base probability is varied. For example, if the predicted probability of a complicated post-operative recovery was 40% for a patient with a given MELD-Na score, the range of probabilities tested in this sensitivity analysis would be 20% to 60%.*

© 2022 Mahmud N et al. *JAMA Network Open.*

eFigure 7. Probabilistic Sensitivity Analyses Using Monte Carlo Simulations*

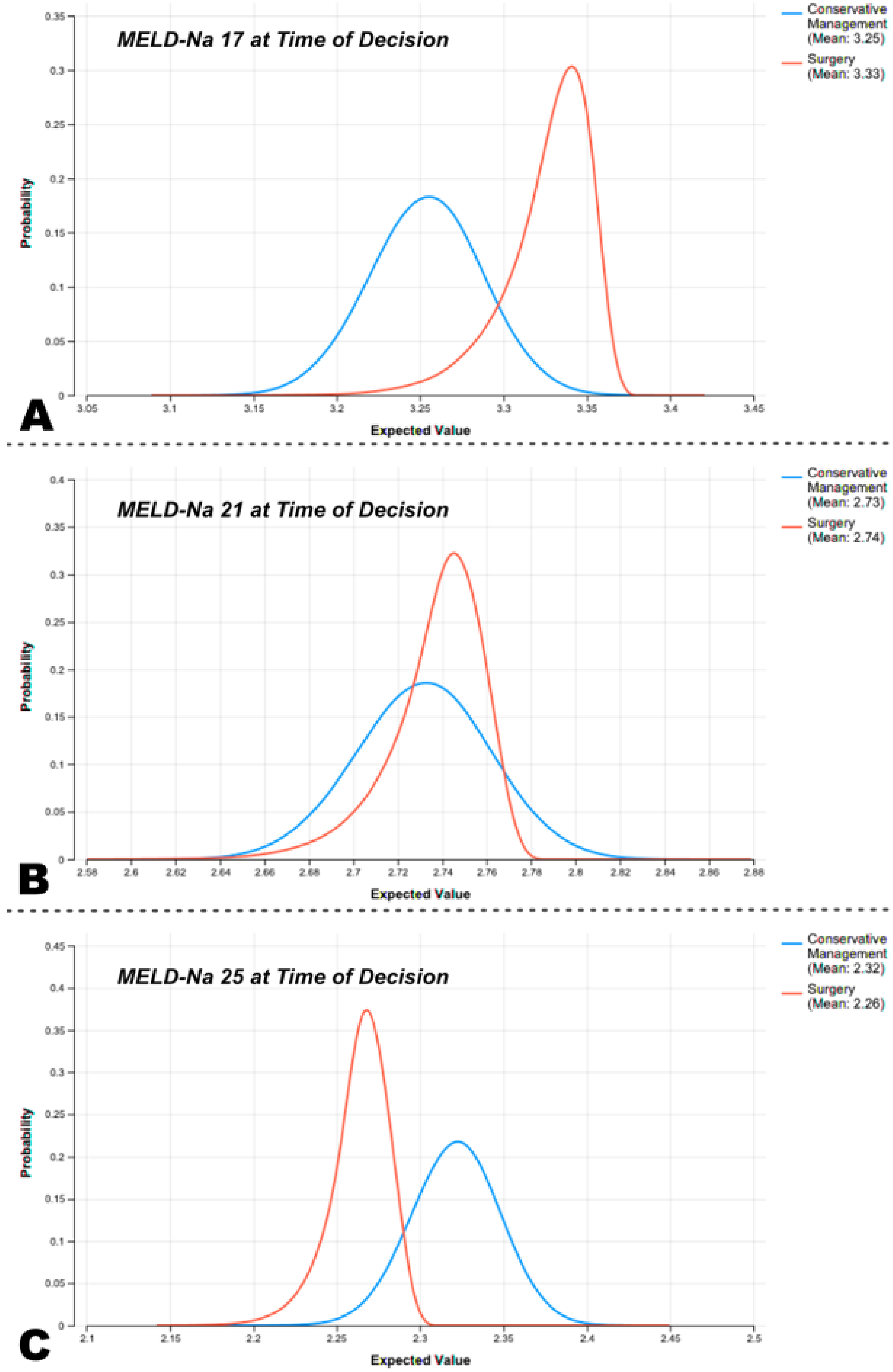

** In probabilistic sensitivity analyses (allowing key model inputs to vary simultaneously), the expected QALYs for surgical management are higher than non-surgical management at MELD-Na 17, but lower than non-surgical management at MELD-Na 25. At an initial MELD-Na 21, the two strategies are near equivalent in terms of expected QALYs.*

© 2022 Mahmud N et al. *JAMA Network Open.*
